# Outbreaks of Human Metapneumovirus in Two Skilled Nursing Facilities — West Virginia and Idaho, 2011–2012

**Published:** 2013-11-22

**Authors:** Sherif Ibrahim, Melissa Scott, Danae Bixler, Randi Pedersen, Jennifer Tripp, Kris Carter, Dean Erdman, Eileen Schneider, Carla Britton

**Affiliations:** West Virginia Bur for Public Health; Southwest District Health, Idaho; Idaho Dept of Health and Welfare; Div of Viral Diseases, National Center for Immunization and Respiratory Diseases; EIS Officer, CDC

During January and February 2012, state and local public health agencies in West Virginia and Idaho, with assistance from facility staff members and CDC, investigated outbreaks of unexplained respiratory illness characterized by high proportions of lower respiratory tract infections (LRTIs) at two skilled nursing facilities (SNFs). Investigations were conducted to determine the extent and etiology of each outbreak and make recommendations to prevent further spread. During both outbreaks, influenza was initially suspected; however, human metapneumovirus (hMPV) was identified as the etiologic agent. Among 57 cases of respiratory illness from both facilities, 45 (79%) patients had evidence of LRTI, of whom 25 (56%) had radiologically confirmed pneumonia; five (9%) had evidence of upper respiratory tract infection (URTI), and seven (12%) could not be classified. Six patients (11%) died. These outbreaks demonstrate that hMPV, a recently described pathogen that would not have been detected without the use of molecular diagnostics in these outbreaks, is associated with severe LRTI and should be considered as a possible etiology of respiratory outbreaks in SNFs.

## West Virginia

On January 5, 2012, an outbreak of respiratory illness among SNF residents was reported to the local health department by an SNF in West Virginia. Clinical and epidemiologic data from ill residents were abstracted from medical records. A case was defined as a respiratory illness in a resident with onset during December 20, 2011–February 20, 2012.

Nasopharyngeal (NP) specimens were sent to a local hospital laboratory for rapid influenza diagnostic tests (RIDT) and to the West Virginia Office of Laboratory Services for influenza real-time reverse transcription–polymerase chain reaction (rRT-PCR) assay. Additional NP specimens were sent to CDC for testing for respiratory pathogens.

The SNF housed 83 residents in a two-wing, single-story building, and employed 95 staff members. Residents shared common dining and activity areas. Cases were identified among 28 (34%) of 83 residents and were distributed throughout the facility. The median age of the 28 patients was 84 years (range: 54–99 years); 15 (54%) were women. Comorbidities included chronic heart disease (64%) and dementia (50%). The median duration of illness was 21 days (range: 3–43 days). Cases were classified symptomatically into URTI or LRTI, with or without radiologically confirmed pneumonia (1) (Table 1). One patient had URTI, and 26 (93%) patients had LRTI, of whom 18 (69%) had radiologically confirmed pneumonia; one case could not be classified. Among the 28 patients, four (14%) were hospitalized, and four (14%) patients died, one of whom had been hospitalized. Among 74 (78%) of 95 staff members who responded retrospectively to a questionnaire about respiratory illness experienced during the SNF resident outbreak, 24 (32%) reported symptoms of respiratory infection during the relevant period.

NP specimens from all 14 patients tested were negative for influenza by RIDT (12 patients) or rRT-PCR (two). Blood cultures from five patients were negative for bacterial growth. Nine NP specimens were submitted to CDC for comprehensive testing for respiratory pathogens by rRT-PCR (Table 2) (2). hMPV was detected in six of nine specimens; no other pathogens were detected. Among the six patients in whom hMPV was detected, five had LRTI, of whom three had radiologically confirmed pneumonia (Table 2). Among four patients who died, one had been tested and was positive for hMPV.

## Idaho

On February 8, 2012, an SNF notified Idaho’s Southwest District Health office of a pneumonia cluster among residents. Patient medical and laboratory records were reviewed. A case was defined as new cough onset in a facility resident during January 31–February 29.

The SNF housed 80 residents in a three-wing, single-story building and employed 119 staff members. Residents shared common dining and activity rooms. Cases were identified among 29 (36%) of 80 residents and were distributed throughout the facility. Among the 29 patients, the median age was 84 years (range: 51–97 years); 18 (62%) were women. Among 27 patients with information, 20 (74%) had two or more comorbid conditions, most frequently dementia (59%), diabetes (38%), and chronic renal failure (34%). Among 26 patients for whom information was available, the median duration of illness was 4.5 days (range: 1–14 days). Among 29 patients, four (14%) had URTI, and 19 (66%) had LRTI, of whom seven (37%) had radiologically confirmed pneumonia; six (21%) could not be classified (Table 1). Among 29 patients, five (17%) hospitalizations and two deaths were reported. Eleven (9%) of 119 staff members reported respiratory illness to the SNF infection control nurse during the outbreak period.

Physician-ordered diagnostic tests, including RIDT (eight patients), rapid test for respiratory syncytial virus (RSV) (one), *Legionella* urinary antigen (three), *Streptococcus pneumoniae* urinary antigen (one), and bacterial cultures on bronchoalveolar lavage (BAL) (one), sputum (one), and blood specimens (five) collected 0–7 days after illness onset all were negative; however, among two of the five patients with blood specimens, blood was collected for bacterial culture 4 days after antibiotic therapy was initiated.

NP specimens from nine nonhospitalized ill residents were collected <4 days after illness onset and tested at the Idaho Bureau of Laboratories, where hMPV was identified by multiplex molecular assay. The nine NP specimens and one BAL specimen subsequently were submitted to CDC for confirmatory testing for hMPV by rRT-PCR; hMPV was detected in six specimens. Among the six patients in whom hMPV was detected, all had LRTI, and half had radiologically confirmed pneumonia (Table 2). Of the two patients who died, one patient was tested and was positive for hMPV.

For both outbreaks, infection control measures included isolation of patients; droplet and contact precautions; enhanced environmental cleaning; cessation of group meals, activities, and new admissions; increased emphasis on identification and exclusion of ill employees; and increased emphasis on hand hygiene and respiratory etiquette among residents, staff members, and visitors.

### Editorial Note

The outbreak in Idaho was the first reported caused by hMPV in an SNF in Idaho, and the outbreak in West Virginia was the second reported outbreak caused by hMPV at an SNF in that state (Sherif Ibrahim, West Virginia Bureau for Public Health, personal communication, 2013). hMPV was first identified during 2001 in respiratory specimens collected during the preceding 20 years in The Netherlands (3). hMPV is responsible for an estimated 5%–15% of LRTI hospitalizations among infants and young children, varying geographically and temporally (4). Although seroprevalence of hMPV-specific antibody is nearly 100% among adults, hMPV can cause symptomatic reinfection throughout life, especially among older adults and immunocompromised persons (4). Among adults, risk factors for severe hMPV disease are advanced age and underlying cardiopulmonary disease (4,5). In both outbreaks reported, the median age of patients was 84 years, and one or more comorbid conditions was present among the majority of patients. Among adults aged ≥65 years in Tennessee, hospitalization rates for hMPV infection have been estimated as 22 cases/10,000 person-years (95% confidence interval: 12.1–33.7) (5).

hMPV typically exhibits peak activity during late winter or early spring in temperate climates (4); however, summer outbreaks attributed to hMPV in LTCFs have been reported (6). hMPV surveillance data from the National Respiratory and Enteric Virus Surveillance System (Figure) and from GermWatch* indicate biennial activity peaks. Increased use of multipathogen molecular diagnostic testing has increased identification and awareness of hMPV as an important etiology of upper and lower respiratory infection (2,7).

Among previously reported outbreaks in SNFs attributed to hMPV, attack proportions up to 36% have been reported. Clinical characteristics of illness ranged from mild upper respiratory infection to respiratory failure and death, with reported case-fatality rates of 0%–31% of cases (4,6,7). In the West Virginia and Idaho outbreaks, 26 (93%) of 28 patients and 19 (66%) of 29 patients, respectively, had LRTI. Four residents in West Virginia and two in Idaho died. Median duration of illness varied widely between West Virginia and Idaho. The longer duration of illness observed in West Virginia might be explained by the higher proportion of patients with LRTI and radiologically confirmed pneumonia.

Unlike identification of a viral cause of a respiratory infection in a young child, identification of a viral cause in an older adult is difficult for many reasons, including protean clinical manifestations and lower viral loads in respiratory specimens. Identification of a cause of LRTI is especially difficult. Early clinical diagnosis and early respiratory specimen collection (e.g., 3–4 days after symptom onset) can increase detection of respiratory viruses by molecular diagnostic tests (8).

The incubation period for hMPV is 5–6 days, and transmission likely occurs as a result of direct or indirect contact with infected secretions spread by fomites or through large particle aerosols, similar to other respiratory viruses (9). In addition to recommended standard and droplet precautions for influenza control, SNF infection control measures for hMPV should include contact precautions to prevent transmission by contact with infected secretions and fomites (10). Consistent with CDC’s long-term care facility influenza control guidelines† ill staff members should be excluded from work until at least 24 hours after they no longer have a fever. Ill staff members and visitors likely represent a significant source of community-acquired respiratory viral infection among SNF residents.

What is already known on this topic?

First identified in 2001, human metapneumovirus (hMPV) is believed to be responsible for an estimated 5%–15% of hospitalizations for lower respiratory tract infections among children. In addition, hMPV can cause symptomatic reinfection throughout life, especially among older adults and immunocompromised persons.

What is added by this report?

These outbreaks of hMPV respiratory illness in skilled nursing facilities (SNFs) caused severe lower respiratory disease in >75% of affected patients, with an overall fatality rate of 11% in a population with a high prevalence of comorbidities or advanced age.

What are the implications for public health practice?

Clinicians should consider hMPV infection in the differential diagnosis of illness in patients with respiratory tract infection in SNFs, particularly when clusters of severe unexplained respiratory infections are detected.

Clinicians should be aware of hMPV as a cause of severe respiratory disease in SNFs. Clusters of unexplained respiratory illnesses should be reported to public health agencies. Prompt reporting of clusters, thorough documentation of clinical symptoms, collection of respiratory specimens early in the course of illness, and use of molecular diagnostic methods can help quickly identify outbreak etiologic agents to prioritize and guide infection control measures, treatment, and chemoprophylaxis decisions. Health departments may contact CDC for assistance with laboratory diagnostics or consultation through the CDC Unexplained Respiratory Disease Outbreaks work group.§

## Figures and Tables

**FIGURE:**
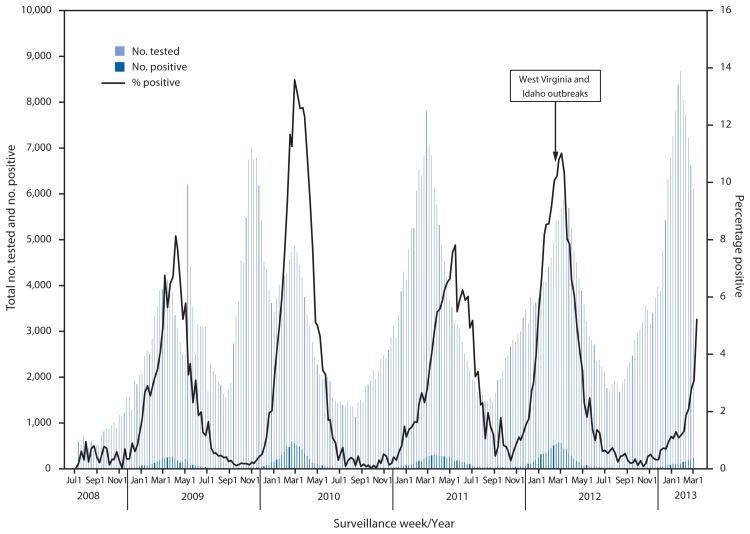
Number of respiratory samples tested and number and percentage of tests positive for human metapneumovirus, by week of report — National Respiratory and Enteric Virus Surveillance System, July 5, 2008–March 2, 2013

**TABLE 1:** Number and percentage of patients with respiratory illness in skilled nursing facilities, by selected signs and symptoms — West Virginia and Idaho, 2011–2012

	West Virginia (N = 28)		Idaho (N = 29)	
		
Signs and symptoms	No.*	(%)	No.*	(%)
Cough	25	(89)	29	(100)
New findings on chest exam/New rales, rhonchi, or wheezes	22	(79)	16	(55)
New or increased sputum/Productive cough	12	(43)	11	(38)
Shortness of breath/Dyspnea	4	(14)	8	(28)
Runny nose/Congestion	3	(11)	4	(14)
Sore throat	1	(4)	1	(3)
Mental status/Functional status changes	1	(4)	5	(17)
Fever >100°F (37.8°C)	11	(39)	7	(24)
Received radiographic imaging	23	(82)	20	(69)
Positive radiographic imaging for pneumonia	18	(78)	7	(35)

* Patient numbers and proportions represent patients for whom this information was documented in medical records. Lack of documentation in medical records does not mean that the signs or symptoms were not present.

**TABLE 2:** Results for specimens tested for human metapneumovirus (hMPV) at CDC from patients in skilled nursing facilities, by infection classification — West Virginia and Idaho, 2011–2012

	West Virginia*			Idaho†		
		
Classification	No. (N = 28)	No. positive	No. tested	No. (N = 29)	No. positive	No. tested
Upper respiratory tract infection	1	0	1	4	0	1
Lower respiratory tract infection	8	2	3	12	2	3
Radiologically confirmed pneumonia	18	3	4	7	4	4
Unclassified	1	1	1	6	0	2

* Specimens confirmed for hMPV by real-time reverse transcriptase–polymerase chain reaction (rRT-PCR). Specimens were screened for 16 viral and seven bacterial pathogens: adenovirus; hMPV; human parainfluenza viruses 1–4; influenza viruses A, B, and C; respiratory syncytial virus (RSV), rhinovirus; human coronaviruses 229E, NL63, OC43, HKU1; enterovirus; *Bordetella pertussis; Chlamydophila pneumoniae; Haemophilus influenzae: Legionella pneumophila; Mycoplasma pneumoniae; Streptococcus pneumoniae*; and *Streptococcus pyogenes*. All specimens were negative for all pathogens other than hMPV.

† Specimens confirmed for hMPV by rRT-PCR at CDC. Specimens were screened at the Idaho Bureau of Laboratories for adenovirus; hMPV; human parainfluenza viruses 1–3; influenza viruses A and B; RSV; entero-rhinovirus by rRT-PCR; viral culture; and qualitative nucleic acid multiplex panel (MP). One specimen from a patient with lower respiratory tract infection was positive for entero-rhinovirus by MP; otherwise all specimens were negative for all pathogens other than hMPV.

## Footnotes

* Available at https://intermountainphysician.org/gw/respiratoryviruses/pages/default.aspx.

† Available at http://www.cdc.gov/flu/professionals/infectioncontrol/ltc-facility-guidance.htm.

§ Available at http://emergency.cdc.gov/urdo.

## References

[1] McGeer A, Campbell B, Emori TG (1991). Definitions of infection for surveillance in long-term care facilities. Am J Infect Control.

[2] Kodani M, Yang G, Conklin LM (2011). Application of TaqMan low-density arrays for simultaneous detection of multiple respiratory pathogens. J Clin Microbiol.

[3] van den Hoogen BG, de Jong JC, Groen J (2001). A newly discovered human pneumovirus isolated from young children with respiratory tract disease. Nat Med.

[4] Kahn JS (2006). Epidemiology of human metapneumovirus. Clin Microbiol Rev.

[5] Widmer K, Zhu Y, Williams JV, Griffin MR, Edwards KM, Talbot HK (2012). Rates of hospitalizations for respiratory syncytial virus, human metapneumovirus, and influenza virus in older adults. J Infect Dis.

[6] Louie JK, Schnurr DP, Pan CY (2007). A summer outbreak of human metapneumovirus infection in a long-term-care facility. J Infect Dis.

[7] Liao RS, Appelgate DM, Pelz RK (2012). An outbreak of severe respiratory tract infection due to human metapneumovirus in a long-term care facility for the elderly in Oregon. J Clin Virol.

[8] Talbot HK, Falsey AR (2010). The diagnosis of viral respiratory disease in older adults. Clin Infect Dis.

[9] Falsey AR, Mandell GL, Bennett JE, Dolin R (2005). Human metapneumovirus. Principles and practice of infectious diseases.

[10] Siegel JD, Rhinehart E, Jackson M, Chiarello L, the Healthcare Infection Control Practices Advisory Committee (2007). 2007 guideline for isolation precautions: preventing transmission of infectious agents in healthcare settings 2007.

